# Deep Learning-Based Quantification of Pulmonary Hemosiderophages in Cytology Slides

**DOI:** 10.1038/s41598-020-65958-2

**Published:** 2020-08-03

**Authors:** Christian Marzahl, Marc Aubreville, Christof A. Bertram, Jason Stayt, Anne-Katherine Jasensky, Florian Bartenschlager, Marco Fragoso-Garcia, Ann K. Barton, Svenja Elsemann, Samir Jabari, Jens Krauth, Prathmesh Madhu, Jörn Voigt, Jenny Hill, Robert Klopfleisch, Andreas Maier

**Affiliations:** 1 Pattern Recognition Lab, Friedrich-Alexander-Universität Erlangen-Nürnberg, Erlangen, Germany; 2 Research and Development, EUROIMMUN Medizinische Labordiagnostika AG, Lübeck, Germany; 3 Institute of Veterinary Pathology, Freie Universität Berlin, Berlin, Germany; 4 VetPath Laboratory Services, Ascot, Western Australia; 5 Laboklin GmbH und Co. KG, Bad Kissingen, Germany; 6 Equine Clinic, Freie Universität Berlin, Berlin, Germany; 7 Department of Neurosurgery, Universitätsklinikum Erlangen, Erlangen, Germany; 8 Institute of Neuropathology, Friedrich Alexander University Erlangen-Nürnberg, Erlangen, Germany

**Keywords:** Image processing, Machine learning, Software, Respiratory tract diseases

## Abstract

Exercise-induced pulmonary hemorrhage (EIPH) is a common condition in sport horses with negative impact on performance. Cytology of bronchoalveolar lavage fluid by use of a scoring system is considered the most sensitive diagnostic method. Macrophages are classified depending on the degree of cytoplasmic hemosiderin content. The current gold standard is manual grading, which is however monotonous and time-consuming. We evaluated state-of-the-art deep learning-based methods for single cell macrophage classification and compared them against the performance of nine cytology experts and evaluated inter- and intra-observer variability. Additionally, we evaluated object detection methods on a novel data set of 17 completely annotated cytology whole slide images (WSI) containing 78,047 hemosiderophages. Our deep learning-based approach reached a concordance of 0.85, partially exceeding human expert concordance (0.68 to 0.86, mean of 0.73, SD of 0.04). Intra-observer variability was high (0.68 to 0.88) and inter-observer concordance was moderate (Fleiss’ kappa = 0.67). Our object detection approach has a mean average precision of 0.66 over the five classes from the whole slide gigapixel image and a computation time of below two minutes. To mitigate the high inter- and intra-rater variability, we propose our automated object detection pipeline, enabling accurate, reproducible and quick EIPH scoring in WSI.

## Introduction

Patients with pulmonary hemorrhage (P-Hem) suffer from repeated bleeding into the lungs, which can result in dyspnea and if untreated, may have life threatening consequences^1^. There are various causes which lead to P-Hem, including drug abuse, premature birth, leukaemia, autoimmune disorders and immunodeficiencies^2–6^. In this paper, we focus on a special subtype of P-Hem called exercise-induced pulmonary hemorrhage (EIPH) in horses. Although EIPH also affects healthy human athletes^7^ and racing greyhounds^8^, it is diagnosed most commonly in racing horses and causes reduced athletic performance^9–12^. The gold standard for diagnosis of P-Hem in humans and equine animals is to perform cytology of bronchoalveolar lavage fluid (BALF)^4,13^ using a scoring system as explained by Golde *et al*.^4^. The red blood cells of the bleeding are degraded into an iron-storage complex called hemosiderin by alveolar macrophages. Hemosiderin-laden macrophages are called hemosiderophages. Prior to microscopic evaluation, the cells are extracted by the BALF procedure and stained with Perlss’ Prussian Blue^14^ or Turnbull’s Blue^15^ in order to visualise the iron pigments contained in the hemosiderin. According to the commonly used scoring system (macrophages hemosiderin score) by Golde *et al*.^4^, alveolar macrophages can be distinguished into five grades depending on their hemosiderin content. This scoring system is based on the principle that a higher score correlates with increased alveolar bleeding^16^.

The macrophages’ hemosiderin score is determined on cytological specimens, which can be digitalised using a whole slide scanner resulting in whole slide images (WSI). One of the main issues with manual counting of hemosiderophages in digital microscopy - just like in traditional light microscopy - is that it is a laboursome and time-consuming task. More importantly, these images are commonly subject to inter- and intra-observer variability. Additionally, there is the problem that the continuous process of hemosiderin absorption is mapped to a discrete grading system. To our knowledge, no previous research has investigated the use of end-to-end, deep learning-based object detection methods for the multi-class problem of pulmonary hemorrhage on WSI. In particular, no study to date has examined the inter- and intra-observer variability for hemosiderophage classification, which is crucial when comparing human performance to algorithmic approaches. This is especially important, since there is no measurable ground truth available and therefore the consistency of the ground truth annotation by an expert is unknown. In this work, the main objective is to develop an overarching deep learning-based system for the analysis of whole slide EIPH images. This includes the detection and classification of hemosiderophages in an accurate, efficient, explanatory and reliable manner.

The major contributions of this paper are as follows: Firstly, we created the largest published data set of fully annotated EIPH images, containing 78,047 single cell annotations by a pathology expert. Secondly, we conducted an analysis of the inter- and intra-observer variability for the classification of single hemosiderophages (CoSH) by multiple experts and compared the results with deep learning-based methods. Thirdly, we developed a custom network architecture dedicated to multi-class whole slide analysis (MCWSA).

This results in a deployable object detection system for EIPH on WSIs, which can process gigapixel images in under two minutes on a modern graphics processing unit (GPU) and is freely available for research purposes.

## Related Work

To date, the topic of hemosiderophage classification and quantification has not been approached using computer vision methods. However, there have been numerous studies in the past decades with the goal of detecting cells, nuclei and mitotic figures for multiple modalities like digital fluorescence microscopy and histopathology^17–19^. Historically, this started as hand-crafted low-level feature extraction^20–22^. With the recent advent of deep learning-based techniques^23^ these methods transitioned into modern end-to-end optimised object detection algorithms like Faster-RCNN^24^, SSD^25^ or RetinaNet^26^. Their underlying end-to-end optimisation approach is the foundation of their success in object detection challenges for natural images like PASCAL VOC^27^ and MS COCO^28^ where no classical approach could outperform a modern deep learning-based object detection method^29^ since 2014. The aim of object detection algorithms is to predict the bounding box as well as a class for multiple objects irrespective of the scale or a partial occlusion of the objects. These methods have generated state of the art results in the fields of pedestrian-, face- and car-detection and are used in state of the art autonomous vehicles as well as the interpretation of satellite images^27,28,30^. Regarding the field of digital pathology object detection, the review by Litjens *et al*.^31^ reveals that no one had implemented deep learning-based object detection methods for the evaluation of medical images as of 2017. In contrast, they mention that sliding window approaches in combination with a deep learning-based classification network or U-Net-like segmentation architectures^32^ are being commonly used. The frequent use of U-Net in particular is quite remarkable since segmentation provides no means of separating touching or overlapping objects and these methods highly rely on post-processing steps for the task of separation. Additionally, in the case of U-Net, the architectures are computationally more complex due to their encoder-decoder architecture. Moreover, these networks require a pixel-wise annotation mask for obtaining better results, which is time-consuming compared to the relatively simple and fast creation of bounding box annotations needed for object detection methods. Ferlaino *et al*.^33^ used deep learning-based object detection on fully annotated multiclass WSI. For this, they employed RetinaNet^26^ for nuclei detection and a separate, not end-to-end trainable, network for nuclei classification.

Modern object detection approaches can be categorised into the two major categories of single stage and two stage algorithms. In single stage setups, the task of detection and classification is solved in one single network, examples are YOLO^34^, SSD^25^ or RetinaNet^26^. In two stage algorithms, the task of detection is solved by the use of a region proposal network (RPN)^24^ in the first stage and then classified using an additional network in a subsequent stage. While two stage detection is more accurate in general, the single stage methods yield the better ratio of accuracy and inference speed^35^. This trade-off between speed and accuracy is crucial when analysing WSI with billions of pixels. In this work, we used RetinaNet as a starting point for analysing EIPH on WSI because its architecture is straightforward, easy to modify and adapt for WSI analysis.

## Material

Our research group built a data set of 17 cytological slides of equine bronchoalveolar lavage fluid. The slides were prepared by cytocentrifugation and stained for iron content with Prussian Blue (n = 10) or Turnbull’s Blue (n = 7) which results in an identical colour pattern. Digitalisation of the glass slide was performed using a linear scanner (Aperio ScanScope CS2, Leica Biosystems, Germany) at a magnification of 400× (resolution: $0.25\frac{\mu m}{px}$). Finally, all macrophages on each slide were annotated by a veterinary pathologist. All bronchoalveolar lavage fluids were obtained from horses with clinical signs of lower respiratory tract disease during routine diagnostic service for therapeutic reasons. Written informed consent was obtained from the owners. Therefore, no animal was harmed for the construction of this data set. Individual case histories were not considered in the present study and all data we received was anonymised by the routine diagnostic service in advance. Using the open source software solution SlideRunner^36^, we were able to build a database that includes the annotations for each hemosiderophage on the slides with their corresponding grade. This was done by first annotating all pulmonary macrophages and afterwards classifying them into their corresponding grade. The scoring system for hemosiderophages was introduced by Golde *et al*.^4^ and consists of five classes: It ranges from zero (no intracytoplasmic blue coloured pigment) to four (cell filled with hemosiderin; dark blue throughout cytoplasm). The final score was calculated by the method of M. Y. Doucet and L. Viel^16^ which is an adaptation of Golde *et al*.^4^ to be used for horses. In this scoring system, three hundred alveolar macrophages were first graded from zero to four, then the total number per grade was divided by three and multiplied with the corresponding grade. The resulting total hemosiderin score (THS) thus ranges from zero to four hundred. If the score is higher than 75 then the diagnosis pulmonary hemorrhage is considered to be confirmed. The completely annotated data set consists of 17 slides and covers an area of 1,266 mm^2^(mean = 74 mm^2^, SD = 9 mm^2^) containing 78,047 labelled cells (mean = 4,591, SD = 3,389) (see Table 1) making it the largest published data set of hemosiderophages and one of the largest of WSI. This novel data set allows us to perform object detection on whole hemosiderophages slides for the first time.

**Table 1:** Data set statistics for each fully annotated WSI.

File	Staining	Total Cells	Score	Count of Cells by Grade						
				0	1	2	3	4	mean	SD
01_EIPH	Prussian	4446	126	1013	1782	1218	348	85	1.26	0.96
02_EIPH	Prussian	12812	72	5084	6203	1450	64	11	0.72	0.68
03_EIPH	Prussian	6325	37	4295	1697	330	3	0	0.37	0.58
04_EIPH	Prussian	5448	63	2551	2379	508	10	0	0.63	0.66
05_EIPH	Prussian	2489	34	1754	634	99	2	0	0.34	0.55
06_EIPH	Turnbull	2992	41	1908	933	148	3	0	0.41	0.59
07_EIPH	Turnbull	1073	235	48	127	352	495	51	2.35	0.91
08_EIPH	Turnbull	924	67	471	290	160	3	0	0.67	0.76
09_EIPH	Turnbull	4752	216	568	1053	932	1446	753	2.16	1.27
10_EIPH	Prussian	10385	208	592	2131	4037	3098	527	2.08	0.96
11_EIPH	Prussian	5751	59	2839	2452	435	25	0	0.59	0.65
12_EIPH	Turnbull	1112	35	767	302	43	0	0	0.35	0.55
13_EIPH	Turnbull	968	43	637	252	70	8	1	0.43	0.67
14_EIPH	Prussian	3143	39	1995	1062	81	5	0	0.39	0.55
**15_EIPH**	**Prussian**	**1841**	**148**	**283**	**553**	**859**	**131**	**15**	**1.48**	**0.86**
**16_EIPH**	**Prussian**	**6491**	**87**	**2611**	**2509**	**984**	**363**	**24**	**0.87**	**0.89**
**17_EIPH**	**Turnbull**	**7095**	**133**	**1639**	**2566**	**1818**	**1066**	**6**	**1.33**	**0.99**

The columns show the total number of alveolar macrophages/hemosiderophages, the number of cells for each grade and their corresponding mean grade and standard deviation. The three final bold lines indicate the test set.

## Methods

The research was carried out in accordance with the Code of Ethics of the World Medical Association (Declaration of Helsinki) and the guidelines of the institutions conducting the experiments.

The aim of this work was to develop and compare algorithmic approaches for predicting the hemosiderophages score of WSI. In order to assess how challenging the classification of single hemosiderophages (CoSH) is, we investigated two methods considering the single cell labels as a classification and as a regression task. We then compared the results with human performance. Additionally, we present methods for multi-class WSI analysis (MCWSA). Here, we adopted state of the art deep learning-based object detection and regression approaches. We used a support vector regression to draw a baseline. To compensate for the sparse cell distribution, we introduce a novel quadtree-based sampling approach to train the object detection networks.

### Human performance evaluation

In order to compare our algorithmic approaches with human recognition performance, we investigated the accuracy and reproducibility of nine cytology experts in labelling single cell pulmonary hemosiderophages. We divided them into three groups according to their qualification and experience with BAL cytology. Each group contained three participants:

• (E)xpert: Veterinary pathologists or clinician with high degree of experience in BAL cytology.

• (P)rofessional: Professional clinician or pathologist with basic experience in BAL cytology.

• (B)eginner: General skills in cytology, but no experience with BAL cytology in particular.

To evaluate the human inter- and intra-observer variability for single cell classification, we extracted two test sets containing 1,000 cells each. For test set 1, the images were randomly selected among the labelled cells resulting in a representative distribution. Test set 2 contained 1,000 cells with a balanced distribution of 200 cells per grade.

Each of the nine cytology experts was asked to classify two thousand cells from the single cell test set 1 and 2. We did not set a time limit to perform this task. In order to measure the intra-observer variability, they were asked to classify all cells again two weeks after the initial assessment. The participants were instructed to perform classification according to the methods published by Doucet *et al*.^16^.

### Sampling strategy

Taking into account that not all slides contain hemosiderophages of grade three and four, we used the same fourteen slides to train and validate. However, we used the upper half of each image for training and the lower half for validation in order to prevent over-fitting. Three separate slides were selected as hold out test set slides.

For deep neural networks, it is beneficial to be trained with equally distributed labelled examples. As shown in Table 1, cell grade 3 and 4 rarely occur on some of the WSI. For example, slide 14 includes only one grade 4 and eight grade 3 hemosiderophages. This means that with an image size of 35,999 × 34,118 pixels and random sampling with a patch size of 1024 × 1024 pixels, the chance to sample the grade 4 cell is only 0.08% percent.

#### Two-stage cluster sampling strategies

For this sampling strategy, we clustered all cells from one WSI on the basis of their grade. For training, we randomly selected one of those clusters and chose one of the cells within that cluster by chance. Then a patch is randomly shifted in the direct proximity of that cell and the area is sampled for training.

#### Generic quadtree sampling strategies

**Figure 1:**
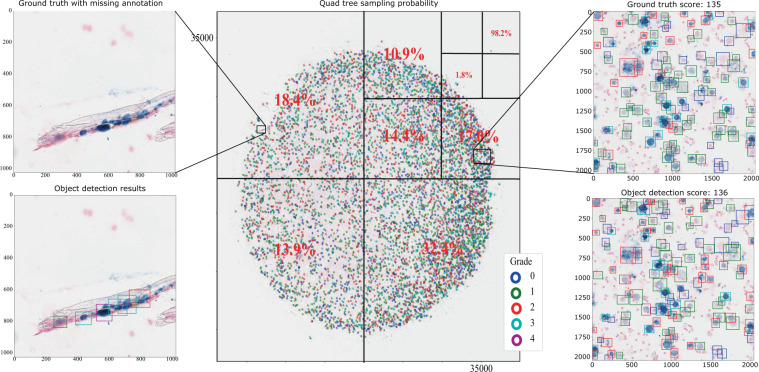
Left: Clumps of hemosiderin in an area with artefacts (hair). The used staining method is inadequate to distinguish between intra-cellular and extra-cellular hemosiderin, clearly making the annotation of the area especially ambiguous. Centre: Example for the sampling strategy on image 17_EIPH Turnbull blue with 7,095 cells. We can see a high sampling probability for the node with the only grade four cell. Each cells was marked as a dot. Right: Object detection result for a region of the image 17_EIPH Turnbull blue with their ground truth on top and the predictions at the bottom.

We developed a novel sampling strategy for microscopy images based on a quadtree in order to consider the probability of occurrence of cells as well as their neighbouring cells (see Fig. 1 center). At each level of the quadtree (depth of the tree can be customised), we saved the cells, their corresponding sampling probability and their grade. As seen in Fig. 1 (center), at each level of the quadtree, we have up to four nodes. One constraint for the tree while it is being created was that there must be at least three hundred cells in each node. One other option would be that the size of the final node must be identical to the training patch size (e.g. 1024 × 1024 pixels). In contrast to the sampling strategy described in the previous section, we can sample at nodes without any cells by defining a minimum probability. To train our networks, we created a quadtree with a depth of three. To create a training sample, we randomly traversed the quadtree according to the sampling probability of the cells. Figure 1 visualises this novel sampling approach. At the first level, the image is divided into four nodes with the sampling probabilities of 35.3%, 32.4%, 13.9% and 18.4% (clockwise). In this example, the top right node was selected by chance and was traversed further. This process was repeated until the final node at level three was reached and one patch was extracted for training.

### Single cell classification (CoCH)

The hemosiderophages score is based on a subjective, semi-quantitative method in which each cell in a selected region of the WSI is assigned one out of five grades (ranging from zero to four). However, this quantised grading system does not reflect the biological nature since there is a continuous gain of iron in the hemophages as opposed to a stepwise rise. To take this continuous increase into account, we propose a regression-based cell score estimation. We then compare the result to the classification approach mimicking the human scoring system.

#### Classification

For the cell-based classification task, we used a compact ResNet-18 Architecture^37^ pre-trained on ImageNet^38^ with a fully connected two layer classification head and a final softmax activation. The cells used for training and validation were extracted according to the proposed quadtree-based sampling strategy. The Network was trained in two stages with the Adam optimiser and a maximal learning rate schedule of 0.01. Categorical cross entropy was used as the loss function. First, we trained only the classification head for three epochs, afterwards we fine-tuned the complete network for an additional twenty epochs until convergence was reached.

#### Regression

**Figure 2:**
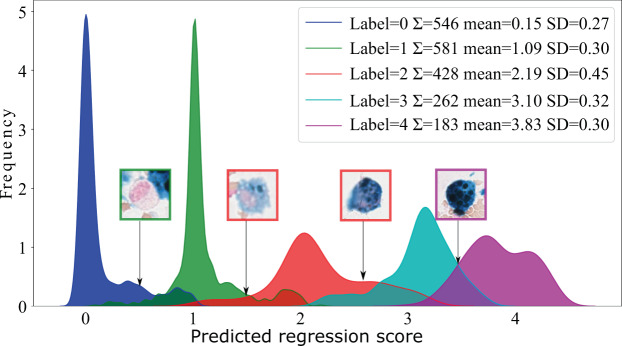
Cell-based regression results on the test data set visualised as a density histogram for the predicted scores. As an example, both cells in the middle are labelled with grade two and the regression model assigned very different scores to both, which is also clearly comprehensible from the visual appearance of the cell.

As stated, the hemosiderin absorption is a continuous process which is mapped to a discrete grading system. To take his continuity into account, we developed a network with a regression head and a final scaled sigmoid activation which predicts continuous values in a range of −0.5 to 4.5. This compensates for the implementation instability for sigmoid activations close to zero and one. The main focus of the experiment was to estimate the intra-grade confusion and increase the human interpretability of the results. This modification enables the network to predict decimal values between any two grades given that the cell has features supporting two grades, which is not possible with a classification approach (see Fig. 2). The network and training schedule were applied as described in the single cell classification paragraph. The mean squared error was used as the loss function.

### Object detection-based WSI score estimation (MCWSA)

**Figure 3:**
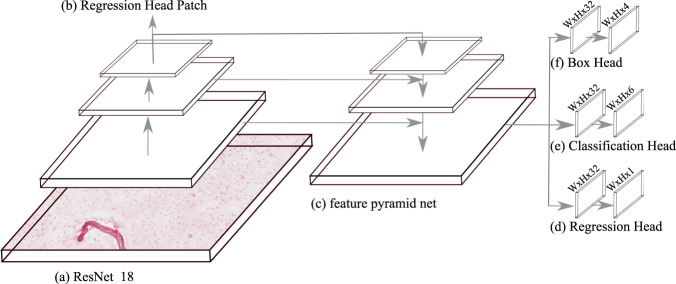
Object detection and score prediction based on RetinaNet. (**a**) ResNet-18 is used as input network for the (**c**) Feature Pyramid Network^39^ to generate rich, multi-scale features. The features ResNet-18 extracted from the patch are used for a direct regression-based score estimation. (**d**) Predicts a regression-based score for each cell, (**e**) classifies the cell into the five grades and background. (**f**) Is used for regressing from anchor boxes to ground truth bounding boxes.

Besides investigating pure classification performance on single cells where the coordinates are previously known, the actual task in diagnostics is the estimation of scores on complete WSIs or subparts thereof. Object detection networks mimic human expert behaviour by both detecting and classifying the cells and calculating the score afterwards. One object detection approach with a good accuracy-speed trade-off is RetinaNet^26^ which is a single, unified network composed of a backbone network for feature extraction (see Fig. 3a). A feature pyramid network (FPN)^39^ is built on top of the feature extractor to generate rich, multi-scale features by combining low-resolution with semantically strong features and high-resolution with semantically weak features (see Fig. 3c). On each layer of the FPN, a classification subnet and a regression subnet are called to make predictions (see Fig. 3d,e). The classification head predicts the probability of the target object’s presence at each spatial position for each anchor. Anchors are defined by the scale and aspect ratio to match the targeted objects on each spatial position. To compensate for the class imbalance, focal loss^26^ was employed during training. The bounding box regression subnet (see Fig. 3f) is generally built in a similar fashion as the classification head but was trained with smooth L1 loss and predicted four coordinates (x-offset, y-offset, width, height) for each box if a corresponding anchor box existed.

We have modified the RetinaNet architecture in three significant ways to further optimise it for hemosiderophage WSI analysis. Firstly, we added an additional regression head which predicts the hemosiderophages score for each hemosiderophage (see Fig. 3f). This had the intent to increase the human interpretability of the results. As the loss function for the cell-based regression head, mean squared error was used. Secondly, to utilise the features extracted from the RetinaNet backbone, we fitted an additional regression head on top of the ResNet-18 feature extractor for patch-wise hemosiderophages score prediction. This process is further described in the later section *deep learning-based regression* and visualised in Fig. 3b. Mean squared error was used as loss function for the patch-based regression head. The total loss for training our network was calculated by Eq. 1, where *c* specifies the ground truth grade, $\gamma$ is a tuneable focusing parameter, ${\alpha }_{t}$ the class imbalance weighting factor, ${p}_{t}$ is the model’s estimated probability for the class with grade c = 1, and x,y are the arbitrary shapes. The network was trained with the Adam optimiser by using a maximal learning rate of 0.001 for 100 epochs until convergence was reached. Additionally, to minimise the number of anchors and therefore further optimise the architecture towards inference speed we only used the 32 × 32 feature map from the FPN. This was motivated by the fact that anchors of higher feature map sizes did not fit the small cell sizes and are limited in their total number.

1 $$\begin{array}{ll}{\rm{TotalLoss}}\,(x,y,{p}_{t},c)\,= & -{\alpha }_{t}{(1-{p}_{t})}^{\gamma }\,\log ({p}_{t}))\\  & +\frac{1}{n}\mathop{\sum }\limits_{i}^{n}\,\{\begin{array}{ll}0.5{({x}_{i}-{y}_{i})}^{2}, & {\rm{if}}\,|{x}_{i}-{y}_{i}| < 1\\ |{x}_{i}-{y}_{i}|-0.5, & {\rm{otherwise}}\end{array}\\  & +\frac{1}{n}\mathop{\sum }\limits_{i=1}^{n}\,{({c}_{i}-{\hat{c}}_{i})}^{2}\\  & +\frac{1}{n}\mathop{\sum }\limits_{i=1}^{n}\,{({c}_{i}-{\hat{c}}_{i})}^{2}\end{array}$$

For comparison, we additionally tested Faster-RCNN^24^ with a ResNet-50 backbone and SSD^25^ with MobileNetV2 as provided by Huang *et al*.^35^. Both networks were trained with the Adam optimiser and a learning rate of 0.0001 for 100 epochs until convergence was reached. All networks were trained with random rotation, horizontal and vertical flips, but without intensity augmentations. This was appropriate since a shift in intensity could alter the cell grade.

### Estimation based on image patch regression

Direct estimation of the hemosiderophages score by using an image patch-based regression approach is an alternative if the bounding box illustration is not required. Furthermore, an image patch-based regression approach could be used to find regions of interest efficiently even with standard computer vision approaches which we will discuss in the following two methods for a regression-based score estimation. While the first one used a support vector machine (SVM), the second was an adaptation of the RetinaNet architecture. The goal of the regression-based algorithm was to predict the grading score in a range the from zero to four on an image patch and to average the results for a total WSI.

#### Support vector machine

In order to set a computationally inexpensive baseline for the task of estimating a hemosiderophages score, we trained a support vector machine with a Radial Basis Function (RBF) kernel and a convexity value of 0.1. These parameters were found by a grid search for the kernel and complexity parameter. As features we used the extracted histograms of a hundred patches per WSI with the sampling strategy described before.

#### Deep learning-based regression

To estimate the hemosiderophages score with a deep learning-based method we used the features extracted from RetinaNet and added two fully connected layers and a sigmoid activation for the regression head (see Fig. 3b). To compensate for the numerical instability of sigmoid activations close to zero and one and in order to enable a prediction score of up to grade four we scaled the sigmoid activation to a range from −0.5 and 4.5. The deep learning-based regression network was trained as a part of our RetinaNet-based object detection pipeline described in section object detection-based WSI score estimation.

## Results

All experiments were run on a Linux workstation with a NVIDIA Quadro P5000 graphics card. The average calculation time for the object detection task was 101 seconds per WSI. The code for all experiments is available online and implemented in pytorch^40^ with fast.ai. The trained model can be downloaded freely and utilised with the open source software SlideRunner^36^ as shown in the Supplementary Video file.

### Object detection evaluation

*Average Precision* (AP) was originally introduced in the 2007 PASCAL VOC challenge^27^ and is commonly used to assess object detection performance. AP is the average detection precision under different recalls and mean Average Precision (mAP) is the average over all five grades.

### Cell classification (CoCH)

**Figure 4:**
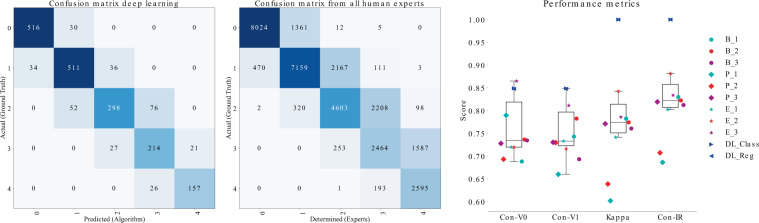
From left to right: Confusion matrix for the automatic single cell classification results; Accumulated confusion matrix for all human experts; On the right the performance metrics diagram visualise the results for the concordance with the ground truth for trial one and two (Con-V0, Con-V1). Additionally, the intra-rater concordance (Con-IR) and Cohen’s Kappa are shown. [B = Beginner, P = Professional, E = Expert, DL = Deep Learning approach.].

**Figure 5:**
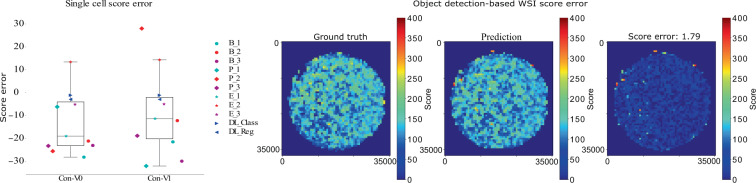
The left diagram visualises the regression error for the single cell classification task. The three remaining figures show the object detection results from test set (slide #17) on 1049 patches of size 1024 × 1024. Ground truth (left), predictions (middle) and error (right). Large errors appearing at the outer circle boundary can be explained by missed cell annotations.

As stated above, we conducted an assessment of expert classification performance for comparison and to set a baseline. Comparing human experts and the deep learning classification pipeline, we found only offsets by one class by the deep learning system, whereas human expert disagreement was generally higher, especially for the higher grades 2, 3 and 4. In these categories disagreement in grade was significant for some cases (see Fig. 4). Concordance with the ground truth data was 85% for both automatic methods, whereas the human experts scored in a range of 69–86% (mean = 74, SD = 5) for the first round of labelling (V0) and 66–81% (mean = 73, SD = 4) for the second round of labelling (V1). This illustrates that we were able to reach human expert-level concordance with the cell-based regression and classification approach. The intra-observer variability ranges from 68 to 88% (mean = 79, SD = 6) with a mean Cohen’s kappa score of 0.74. The inter-observer Fleiss’ kappa score was 0.67 at the first round of labelling (V0) and 0.68 at the second (V1). For the first round of labelling (V0) the F1 score per grade was F1(0) = 0.86 (SD = 0.08), F1(1) = 0.74 (SD = 0.08), F1(2) = 0.62 (SD = 0.11), F1(3) = 0.50 (SD = 0.16) and F1(4) = 0.68 (SD = 0.21) and the second round of labelling (V1) F1(0) = 0.87 (SD = 0.07), F1(1) = 0.73 (SD = 0.07), F1(2) = 0.60 (SD = 0.09), F1(3) = 0.47 (SD = 0.14) and F1(4) = 0.61 (SD = 0.28). The process of classifying two thousand cells took each expert roughly two hours while the deep learning approach took five seconds. The human expert classification accuracy lead to a hypothetical mAP in the range of 0.57 (concordance 0.68) to 0.74 (concordance 0.86) with a mean of 0.60 (concordance 0.73) under the precondition that all cells are detected exactly as in the ground truth. The ground truth mean hemosiderophages score for the 2000 cells was 147 which was predicted by both deep learning approaches with a margin of 1 whereas the human experts have a mean score error of −15 with a standard deviation of 12. The results are visualised in the left sub figure of Fig. 5.

### Object detection (MCWSA)

Our object detection approach showed a mean average precision (mAP) of 0.66 (SD = 0.18, IoU = 0.5) over the three test set WSIs with a total of 3,518 patches and 15,427 cells. Table 2 shows the results per WSI over all tested networks with a maximal mAP of 0.66 reached by multiple approaches. The average error for cell-based grade score was 9 (SD = 24) and was calculated by taking the absolute difference of all ground truth cell grades and the predicted grades. For better understanding, a patch-wise analysed WSI is shown in Fig. 5.

**Table 2:** Comparison of multiple object detection architectures with their corresponding backbone, number of parameters, accuracy, score error and average inference speed per test WSI.

Architecture	Backbone	Parameter	mAP_50	Score Error	Inference speed
Ours	RN-18	11.434.555	0.64	15	101s
Ours	RN-18	11.987.739	0.65	13	101s
Ours	RN-18	13.683.675	0.66	9	103s
Ours	RN-18	22.625.439	0.66	9	111s
RetinaNet	RN-18	19.729.755	0.66	9	111s
RetinaNet	RN-34	29.837.915	0.66	9	142s
RetinaNet	RN-50	36.288.347	0.66	8	258s
SSD	MobileNetV2	13.871.354	0.61	21	105s
Faster-RCNN	RN-50	128.383.642	0.66	7	305s
SVM	RBF-Kernel	/	/	21	65s
DL-Regression	RN-18	11.704.897	/	19	92s

We incrementally increased the number of channels and convolutional layers in our implementation until the accuracy converged against 0.66. Additionally, the errors of the deep learning-based regression and of the support vector machine are shown for comparison.

The comparison of the three sampling strategies revealed a good overall convergence for the two stage cluster sampling strategy (mAP 0.66) and the quadtree sampling strategy (mAP 0.66), while completely random sampling showed very slow convergence to a lower mAP of 0.28.

### Patch regression

As stated before, we evaluated two approaches to predict the grade score directly without additionally predicting bounding boxes and compared the results with our object detection-based approach and the ground truth. The bounding box-based approach produced the best results with an error of 9 compared to the deep learning-based regression approach with 19 and the classical support vector-based method with 21 as shown at the bottom of Table 2.

## Discussion and outlook

We demonstrated that the task of classifying hemosiderophages into the corresponding grading system as proposed by Golde *et al*.^4^ is not only monotonous and time-consuming but also highly subjective. This was shown by the observed high inter- and intra-observer variability and a moderate inter-observer reliability of agreement which strongly suggests that a discrete grading system has its limitations for the quantification of pulmonary hemosiderophages. This is an interesting topic for future work. Additionally, human experts who showed a tendency towards assigning grades below the reference grade were occasionally off by two grades. On the other hand, there was no obvious difference between the performance of the three defined groups of participants with different degree of experience with BAL cytology. In this paper, we proposed a single cell-based classification and regression system (CoCH) with a performance comparable to human experts in order to overcome this grading limitation. In contrast to the human experts, the classification and regression approaches showed both plausible and reproducible outcomes while having an extremely high processing speed. However, the CoCH algorithm has the limitation that hemosiderophage cells had to be annotated by a human expert for further classification. Unfortunately, there is currently no true gold standard method such as chemical measurement of iron content which, of course, would be highly beneficial to validate our deep learning methods^13^.

Since manual scoring of P-Hem has some limitations, we proposed the use of computerised quantification. This could lead to a scoring with promising results regarding accuracy, reproducibility and inference speed. We have shown that even with a perfect detection rate at a human level classification, the mAP is less than 0.74. Based on this data set, this defines an upper limit for human and algorithmic approaches, which was almost reached by the streamlined object detection pipeline based on the RetinaNet-Architecture (MCWSA). Patch-based regression approaches did not achieve the accuracy of object-based methods as a consequence of their susceptibility to blue coloured artefacts. The introduction of the quadtree-based sampling strategy led to more stable and better results at the beginning of the training process but ends up with results similar to the two stage cluster-based sampling method. Furthermore, besides the investigated intra-observer variability for single cell classification, we identified regions where the expert and the algorithm yielded different results. These differences could be attributed to artefacts in the sample like hairs (see Fig. 1). The nuclear fast red (counterstain) dyes the nucleus light red but not the cytoplasm, which induces difficulty recognising cell borders. To reduce the possibility that regions of the WSI were missed by the annotating human expert, an interactive augmented annotation method that was trained on already annotated WSI could be introduced. This interactive annotation process could further increase the quality of annotated WSI by highlighting areas of the WSI where human annotations and the deep learning-based predictions strongly diverge (Fig. 5). Furthermore, this interactive annotation method could be used to decrease the amount of required human interactions for annotating WSI by creating a preliminary result which has to be subsequently reviewed by the experts. This process should be closely monitored in order to refrain from introducing a bias towards accepting the deep learning-based predictions and further research is required regarding reliability.

The variance of the human P-Hem scoring could be even higher if human experts have to select a region of interest from the WSI to grade instead of getting single cut-out cells as similar research shows for the task of mitotic count^41^. We can see from Fig. 5 that the score is not equally distributed over the whole image and thus the final score highly depends on the selected region of interest.

Finally, this work has some limitations that need to be mentioned. All ground truth annotations were made by a single veterinary pathologist, data was collected at one laboratory and specimens were digitised with a single slide scanner. The data set comprises only seventeen WSIs, so our proposed approaches need to be validated on a larger, more diverse data set. Furthermore, we have taken no action to make an external colour calibration of the participants’ screens which could positively influence the results of the participants but does not correspond to current clinical practice. In further work, we plan to analyse the effect of manual region selection by human experts and evaluate and reduce its impact on the proposed object detection pipeline. Furthermore, we are going to introduce an interactive annotation to increase the quality of the data set and effectively label new WSIs while analysing possible bias introduced by this. Also, it would be of high interest for us to analyse and evaluate our proposed methods and trained models on human pulmonary haemorrhage data sets. We believe a transfer to an application on human samples may be possible using only a small data set and transfer learning.

## Supplementary information

Supplementary Video.

## Data Availability

The data sets generated during and/or analysed during the current study are available from the corresponding author on reasonable request.
